# Perinatal Hypoxic-Ischemic Encephalopathy Among a Large Public Hospital Population

**DOI:** 10.1001/jamanetworkopen.2024.44448

**Published:** 2024-11-04

**Authors:** Lina F. Chalak, Lynn Bitar, Srinivas Kota

**Affiliations:** Division of Neonatal-Perinatal Medicine, Department of Pediatrics, University of Texas Southwestern Medical Center, Dallas.

## Introduction

Perinatal asphyxia remains a major cause of morbidity and mortality in newborns worldwide, with an estimated 1 to 2 million cases annually.^1^ Despite advancements in maternal-fetal and neonatal care, recent studies have reported a paradoxical rise in perinatal hypoxic-ischemic encephalopathy (HIE), although these studies lack detailed assessments of disease severity.^2,3^

## Methods

The Neurological Neonatal Intensive Care database at Parkland Health, Dallas County public hospital captures detailed data from one of the largest inborn delivery services in the US, with 12 000 deliveries annually. This prospective cohort study was approved by the University of Texas Southwestern’s institutional review board with a waiver of informed consent because data were deidentified. The study includes detailed Sarnat scoring by certified physicians, documenting the severity including mild HIE (using AAP diagnostic criteria). Self-reported race was analyzed in this study because it was included in the maternal characteristics database. We report the incidence rate (IR) of HIE, calculated per 1000 live births (>35 weeks), trended from 2012 to 2023, adhering to Strengthening the Reporting of Observational Studies in Epidemiology (STROBE) reporting guidelines. Depending on the normality of continuous variables, we compared groups with 1-way analysis of variance or Kruskal-Wallis test. The assumption of normality was assessed using the Shapiro-Wilk test. For categorical variables, we used χ^2^ or Fisher exact test. Statistical significance was defined as a 2-sided *P* value less than .05. All statistical analyses were conducted using R version 4.3.1 (R Project for Statistical Computing). Data were analyzed from January 2012 to December 2023. Further details on methods are available in the eMethods in Supplement 1.

## Results

Of the 141 448 live births, 418 were diagnosed with HIE (IR, 2.9). Among those with HIE, 187 newborns (44.7%) received therapeutic hypothermia (TH) for moderate and 39 (9.3%) for severe HIE, while 192 (45.9%) had mild HIE and were not cooled (Table). Joinpoint regression analysis revealed a significant annual percentage change (APC) of 6.34 (95% CI, 1.31 to 11.62; *P* = .02) in the overall cohort (Figure). The APC for mild HIE was significantly increased at 8.95 (95% CI, 4.07 to 14.06; *P* = .01). In contrast, no significant differences were observed for moderate (APC, 3.64; 95% CI, −2.29 to 9.94; *P* = .21) or severe (APC, 5.40; 95% CI, −1.17 to 12.41; *P* = .09) HIE. We found significant differences in the sex distribution, with 236 male patients (56%) and 182 female patients (44%), compared with infants without HIE ($\chi_{1}^{2}=4.44$; *P* = .04).

## Discussion

Our large inborn population study confirms that the changing incidence of HIE is primarily due to improved documentation of mild HIE. Although the overall number of HIE cases has increased throughout the study period, this is mainly attributed to reporting mild HIE.

This study addresses conflicting prior reports of rising HIE rates recently observed in some UK and US studies.^2,3^ These trends may have resulted from off-label TH, the standard of care for moderate and/or severe HIE, conveniently used as a proxy for diagnosis of HIE in hospital databases. Another factor is the complex dynamic nature of neonatal encephalopathy, with overlapping causes needing detailed examinations often lacking in most databases. Other causes of fetal compromise, including preeclampsia, chronic intrauterine hypoxia, and secondary infections inadvertently attributed to HIE, may explain the higher rates seen in some cohorts. By adhering strictly to evidence-based TH^4^ guidelines and accurately defining mild HIE,^5^ we confirm in our cohort that the incidence, diagnosis, and TH treatment for moderate and/or severe HIE have remained unchanged over the last decade, with sex differences of higher incidence in male patients consistent with prior publication.^6^ Reassuringly, the proportion of death, moderate, and/or severe HIE was significantly lower than hypothermia trials published before 2005.

Strengths of this study include detailed neurological examination, universal population-based cord gas screening for fetal acidosis, and validation for data quality and accuracy where data were entered by the same abstractor over the entire period. All deliveries were captured, and no eligible infants missed TH. However, observed trends may differ in transfer centers or other regions. Other limitations include a single delivery site and the observational study design limiting causal inference.

## Conclusions

Overall, this large inborn Texas population study sheds light on the dynamic landscape of therapeutic interventions for HIE and the need for further research to determine the optimal use of TH in mild HIE cases. Furthermore, it suggests that relying solely on TH receipt may not be an adequate marker to assess changes in HIE incidence over time.

## Supplementary Material

Supplement 1 SUPPLEMENT 1.
eMethods

Supplement 2 SUPPLEMENT 2.
Data Sharing Statement

## Figures and Tables

**Figure.**
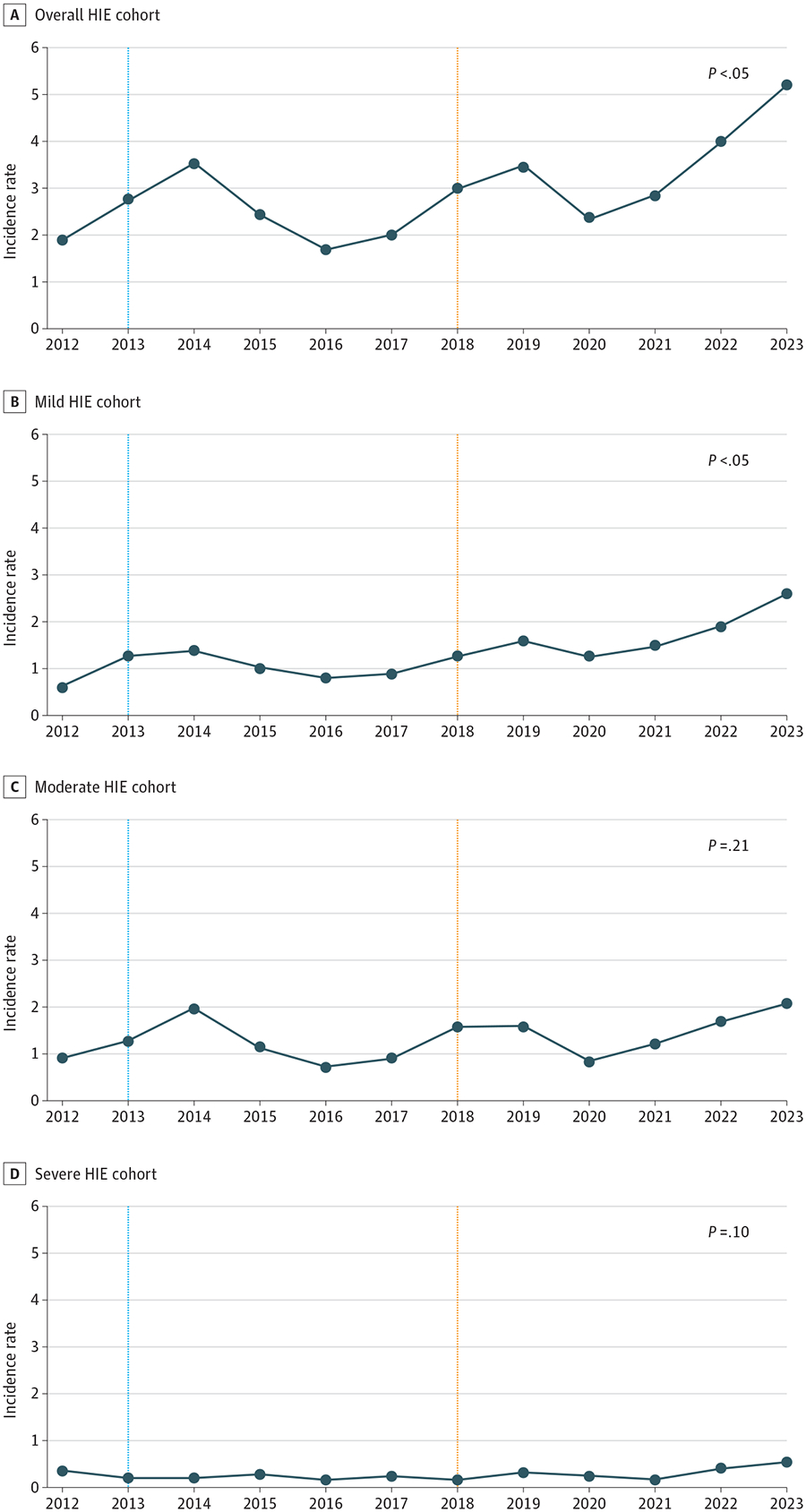
Population Incidence of Perinatal Hypoxic Ischemic Encephalopathy (HIE), 2012 to 2023 Blue line indicates the publication of optimizing cooling and late hypothermia trials,^4^ and orange line indicates when mild HIE was formally defined in the first 6 hours of life in the Prospective Research on Infants with Mild encephalopathy (PRIME) study.^5^

**Table.** Neonatal and Maternal Demographics^a^

	Participants, No. (%)			
		HIE severity		
Characteristics	Overall cohort (N = 418)	Mild (n = 192)	Moderate (n = 187)	Severe (n = 39)
Neonatal				
Sex				
Female	182 (43)	80 (42)	82 (43)	20 (51)
Male^b^	236 (57)	112 (58)	105 (57)	19 (49)
Gestational age, median (IQR), wk	39 (38–40)	39 (38–40)	39 (38–40)	39 (37–40)
Race				
Asian	14 (3)	8 (4)	6 (3)	0
Black	96 (23)	41 (21)	47 (25)	8 (20)
White	307 (73)	143 (74)	133 (71)	31 (79)
Ethnicity				
Hispanic	286 (68)	133 (69)	122 (65)	31 (79)
Non-Hispanic	132 (32)	59 (31)	65 (35)	8 (20)
Birth weight, mean (SD), g	3271 (623)	3260 (589)	3271 (654)	3325 (648)
Head circumference, median (IQR), cm	34 (33–35)	34 (33–35)	34 (33–35)	34 (33–35)
Apgar score, median (IQR)				
1 min^b^	2 (1–4)	3 (2–4)	2 (1–3)	2 (1–3)
5 min^b^	6 (3–7)	6 (5–8)	5 (2–6)	4 (2–7)
10 min^b^	7 (4–8)	7 (6–8)	6 (4–7)	6 (3–7)
Seizures^b^	77 (18)	13 (7)	52 (28)	12 (31)
Days of life at discharge, median (IQR)^b^	11 (6–20)	7 (5–11)	15 (10–26)	16 (11–27)
Death^b^	26 (6)	3 (2)	20 (11)	3 (8)
Maternal				
Maternal age, median (IQR)	27 (22–34)	27 (23–34)	28 (22–33)	25 (22–36)
Chorioamnionitis	112 (27)	53 (28)	47 (25)	12 (31)
Diabetes	56 (13)	30 (16)	20 (11)	6 (15)
Preeclampsia	127 (30)	54 (28)	59 (32)	14 (36)
Prenatal care	402 (96)	188 (97)	179 (96)	35 (92)
Gravidity, median (IQR)	2 (1–4)	2 (1–4)	2 (1–4)	2 (1–3)
Parity, median (IQR)	1 (0–2)	1 (0–2)	1 (0–2)	0 (0–2)
Vaginal delivery	131 (31)	63 (33)	55 (29)	13 (33)

a When the normality assumption was violated, median and IQR were reported, and the Kruskal-Wallis test was used to assess statistical significance.

b Indicates statistical significance (*P* < .05).

## Data Availability

See Supplement 2.
