# Supplementary material for: Perinatal Hypoxic-Ischemic Encephalopathy Among a Large Public Hospital Population
Source: JAMA Netw Open. Author manuscript; Available in PMC 2024 Nov 18. (PMC11555543; doi:10.1001/jamanetworkopen.2024.44448)
Supplement: Supplement 1 — eMethods [file NIHMS2035340-supplement-Supplement_1.pdf]

Supplemental Online Content

Chalak LF, Bitar L, Kota S. Perinatal hypoxic-ischemic encephalopathy among a large public hospital population. *JAMA Netw Open*. 2024;7(11):e2444448. doi:10.1001/jamanetworkopen.2024.44448

eMethods

This supplemental material has been provided by the authors to give readers additional information about their work.

© 2024 Chalak LF et al. *JAMA Network Open*.

eMethods

The neurological assessments are conducted by the attending neonatal intensivist in house who is certified yearly by Dr. Lina Chalak using videos and 1:1 hands-on following power point presentation refresher to grade the severity of hypoxic ischemic encephalopathy (HIE) using standardized methods, such as the modified Sarnat exam. This protocol is standardized at this institution, with ongoing training provided on a yearly basis.

Diagnosis for HIE followed the American College of Obstetricians and the American Academy of Pediatrics (AAP) guidelines, which include: (1) the presence of a sentinel event occurring immediately before or during labor and delivery for example a ruptured uterus or severe abruption placentae; fetal heart rate monitor patterns consistent with an acute peripartum or intrapartum event; (2) the presence of fetal acidosis (pH of ≤7.0 or a base deficit of ≥16 mmol/L in a sample of umbilical cord blood or blood obtained during the first hour after birth) and low Apgar scores (a 10-minute Apgar score of <5, or assisted ventilation initiated at birth and continued for at least 10 minutes), (3) evidence of multiple organ injury affecting the heart, liver, or kidneys, (4) neuroimaging with MRI consistent with acute peripartum or intrapartum event and excluding other causes.

© 2024 Chalak LF et al. *JAMA Network Open*.
