# Supplementary material for: Perinatal Hypoxic-Ischemic Encephalopathy Among a Large Public Hospital Population
Source: JAMA Netw Open. Author manuscript; Available in PMC 2024 Nov 18. (PMC11555543; doi:10.1001/jamanetworkopen.2024.44448)
Supplement: Supplement 2 — Data Sharing Statement [file NIHMS2035340-supplement-Supplement_2.pdf]

Data Sharing Statement

Chalak. Perinatal Hypoxic-Ischemic Encephalopathy Among a Large Public Hospital Population. *JAMA Netw Open*. Published November 11, 2024. doi:10.1001/jamanetworkopen.2024.44448

Data
Data available: No

Additional Information
Explanation for why data not available: Data availability statement: Dr. Chalak has full access to all the data in the study and takes responsibility for the integrity of the data and the accuracy of the data analysis. Data access requests can be submitted to Dr. Chalak. Reasonable requests will be considered, and data sharing will comply with relevant data protection and confidentiality protocols.
